# A Hybrid Method Combining Voronoi Diagrams and the Random Walk Algorithm for Generating the Mesostructure of Concrete

**DOI:** 10.3390/ma17184440

**Published:** 2024-09-10

**Authors:** Binhui Wang, Xiaogang Song, Chunying Weng, Xiaodong Yan, Zihua Zhang

**Affiliations:** 1 Department of Civil Engineering, Ningbo University, Ningbo 315211, China; 2211110038@nbu.edu.cn (B.W.); yanxiaodong@nbu.edu.cn (X.Y.); zhangzihua@nbu.edu.cn (Z.Z.); 2 Digital Business School, Zhejiang Business Technology Institute, Ningbo 315012, China; wcybaby2022@163.com; 3 Key Laboratory of Impact and Safety Engineering, Ministry of Education, Ningbo University, Ningbo 315211, China

**Keywords:** mesostructure of concrete, polyhedral aggregate, Voronoi diagram, grading curve, aggregate volume fraction

## Abstract

The modeling of the concrete matrix serves as a foundation for mesoscale analysis of concrete, which provides a crucial avenue for investigating the crack propagation and strength characteristics of concrete. However, the primary prerequisite for conducting such analyses is the generation of aggregate models. By combining the advantages of Voronoi diagrams and the random walk algorithm (RWA), a Voronoi–random walk algorithm is proposed in this paper. The algorithm overcomes the limitations of traditional methods, including constraints on aggregate volume fraction, low computational efficiency, and insufficient randomness in aggregate distribution. The meso-structure of a concrete block was modeled by the proposed method, and then its failure behavior under uniaxial compression was simulated using the finite element method. The numerical results agreed well with the experimental observations, indicating the effectiveness and accuracy of the proposed approach.

## 1. Introduction

Concrete, a widely utilized building material worldwide, is a typical heterogeneous material composed of cement, aggregates, interfacial transition zones (ITZs) surrounding the aggregates, and voids. Due to the relatively low strengths of the ITZs, cracks are prone to emerge in the ITZs and propagate along the profiles of the aggregates. In this sense, the mechanical performance of concrete is closely related to its mesostructure. For a numerical simulation at the mesoscale, the spatial distribution of aggregates should be determined first, and it should meet the grading and packing requirements simultaneously. To date, the approaches for aggregate placement can be divided into four categories as follows.

(1) The “take-and-place” method [1,2,3] involves randomly depositing aggregates into the target domain and then conducting intersection checks to retain aggregates meeting the specified criteria. Although this method ensures rational aggregate grading, it may fall short of achieving a high aggregate volume fraction. For instance, Zhang et al. [4] utilized X-ray computed tomography (XCT) to scan aggregates and employed the take-and-place algorithm to establish mesoscale concrete models.
(2) The “place-and-generate” method [5,6] requires randomly distributed points to be generated within the target domain, and subsequently, various types of aggregates are generated at these points using aggregate growth algorithms [5,7] or Voronoi diagrams [8,9,10]. Although a high aggregate volume fraction can be achieved, the aggregate grading always deviates from the ideal curve.
(3) The “random walk algorithm” (RWA) [11,12] involves sequentially introducing generated aggregate particles into the target domain through a random walk process, including translations and rotations until no more aggregates can be placed. Although the aggregate volume fraction and grading can be ensured, it exhibits a low efficiency in placing polyhedral aggregates. Wu et al. [13] employed the RWA to obtain a mesoscopic model of concrete with convex polyhedra.
(4) The “aggregate packing” method [14] generates aggregates individually and arranges them layer by layer into the domain. While no intersection or overlapping check is required, it may result in a lower aggregate volume fraction due to the presence of gaps between aggregates, and once aggregates are generated by the algorithm, they cannot be moved anymore. Huang et al. [15] utilized XCT to scan real aggregates and employed the aggregate packing algorithm to generate realistic mesoscale concrete models. Liang et al. [16] combined the aggregate packing and take-and-place algorithms to introduce “partial aggregates” into the target domain and established a two-dimensional mesoscopic model for asphalt concrete.

Among the above-mentioned methods for aggregate placement, the Voronoi diagram, which is a place-and-generate method, has garnered significant attention due to its relatively high efficiency. Its application in aggregate placement was initially proposed by Caballero et al. [8]. The fundamental concept involves generating Voronoi cells at seed points through Delaunay triangulation and utilizing them for aggregate generation. Based on this paper, Zhang et al. [17,18] constrained the minimum distance between seed points and controlled the distribution irregularity of Voronoi cells using a random coefficient. Ren et al. [19] uniformly divided the target domain into several sub-domains and altered the shapes of the Voronoi cells by shifting the positions of their centroids. To obtain more realistic models, Naderi et al. [20] transformed Voronoi polyhedra into smooth-surface aggregates using splining techniques. Zhang et al. [21] utilized the XCT technique to obtain real aggregate shape data and employed Voronoi cells as containers for storing aggregates, which could be filled with aggregates smaller than cells. Ma et al. [22] generated coarse aggregates using Voronoi diagrams and scaling procedures and then filled the interstices of the coarse aggregates with fine aggregates using the take-and-place method. Wei et al. [23] proposed a “repartitioning-based aggregate generation” method to repartition the specimen space into Voronoi cells. The aggregates that were consistent with the grading curve were selected so that the grading requirement could be achieved. However, the following drawbacks of the algorithms based on the Voronoi diagram cannot be overlooked. (1) The closely connected aggregates generated by the Voronoi diagrams often fail to adequately represent the mortar phase. (2) Adjacent surfaces of neighboring aggregates frequently appear parallel, deviating significantly from the actual spatial distribution of aggregates. (3) Generated aggregates often struggle to meet grading requirements.

To address the limitations of the algorithms based on Voronoi diagrams, a novel hybrid method for aggregate placement, named Voronoi–random walk (V-RW), is proposed. It utilizes the RWA to generate spherical aggregates that meet grading requirements. The centers of these spherical aggregates serve as seed points for generating a series of polyhedra in the target domain using the Voronoi algorithm. Subsequently, an optimization algorithm is employed to improve the quality of the polyhedra by eliminating sharp angles and short edges, followed by a range mapping algorithm to obtain polyhedral aggregates that satisfy both grading and computational requirements. This approach combines the advantages of the RWA in meeting the grading requirements with the efficiency and high-aggregate-volume-fraction characteristics of the Voronoi algorithm. Additionally, it addresses issues caused by the Voronoi algorithm, such as parallel adjacent faces of neighboring aggregates. As the skeleton of concrete, aggregates have a significant impact on the development of cracks in concrete damage. To verify whether the generated aggregate model can reflect the real situation, numerical simulations were conducted on the mesostructure of concrete under uniaxial compression.

Considering computational efficiency, gradation, and aggregate geometry, this paper proposes a novel aggregate generation algorithm. The computations were performed on an Intel Core i5@4.4 GHz desktop computer with 16 GB of RAM. It requires approximately 1.2 h for a model with a 40% volume fraction of aggregates, with lower volume fractions necessitating less time. The introduced V-RW algorithm generates aggregate models that align with Fuller gradation. In real concrete, the total aggregate volume fraction typically ranges from 60% to 70% [24,25]. By excluding fine aggregates and focusing on coarse aggregates, a model with a 40% volume fraction is acceptable. This paper provides both a theoretical foundation and technical support for subsequent large-scale, multi-scale numerical simulations.

## 2. Voronoi–Random Walk Aggregate Generation Method

### 2.1. Polyhedral Aggregate Generation

In the Voronoi diagram method, the sizes of the generated polyhedra tend to be relatively uniform, which is not conducive to achieving a realistic aggregate grading. As the RWA can obtain reasonable grading curves [12], it is employed to arrange the seed points of the Voronoi polyhedra to achieve good grading. Owing to the advantage of Voronoi diagrams, the preliminary morphology of the aggregates can be provided, reducing the burden of reshaping the aggregates.

#### 2.1.1. Random Walk Algorithm (RWA)

The RWA consists of two main components: aggregate generation and a random walk. First, aggregates that meet the grading requirements are generated within a specified region. Subsequently, these aggregates are gradually introduced into the target domain by a random walk. During the implementation of the RWA, aggregates undergo translation and rotation to simulate the actual pouring and compaction processes of concrete, which include horizontal and downward movements. The aggregates must satisfy two requirements: (1) all aggregates cannot exceed the target domain, and (2) there are no intersections between any two aggregates. The random walk process is executed iteratively until no more aggregates can be added to the target domain. For further details of the RWA, please refer to our previous papers [11,12].

With spherical aggregates as an example, the procedure involves initially determining the target grading and the size of the target domain. Subsequently, spherical aggregates are generated within a specified region and then placed into the target domain by the random walk process. Due to the symmetry of the spherical aggregates, only translation is considered during the random walk process. Throughout the random walk procedure, aggregates are required to adhere to the two aforementioned requirements. The placement of aggregates will be terminated when no new aggregates can be placed into the target domain.

#### 2.1.2. Voronoi Polyhedra Generation Algorithm

Voronoi tessellation can partition a target domain into several sub-domains based on seed points *S_i_*. Each sub-domain, named a Voronoi cell *V_i_*, is defined such that any point *P_i_* within it is closer to its associated seed point than to any other seed point in the set [26], *V_i_* = {*P*|*d*(*P_i_*, *S_i_*) < *d*(*P_i_*, *S_j_*), *i* ≠ *j*}. If there are *N* seed points, then *N* corresponding Voronoi polyhedra can be obtained to represent the tessellation [17].

**Figure 1:**
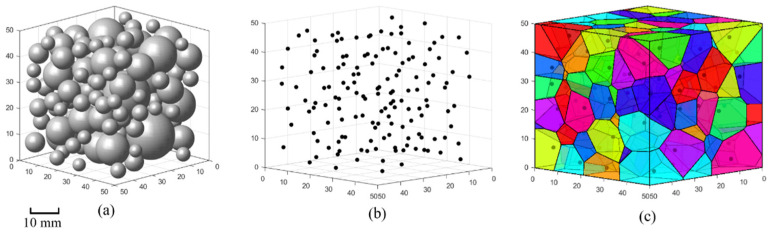
Generation process of Voronoi polyhedra: (**a**) generation of spherical aggregates, (**b**) seed points for the Voronoi tessellation, and (**c**) Voronoi polyhedra generation.

The generation process of Voronoi polyhedra is shown as follows:

(1) Generate spherical aggregates according to the user-defined grading curve.
(2) Employ the RWA to place spherical aggregates into the target domain, as shown in Figure 1a.
(3) Take the centers of the spherical aggregates as seed points for the Voronoi tessellation, as shown in Figure 1b.
(4) Employ the Delaunay triangulation to generate Voronoi polyhedra representing the aggregates based on the generated seed points, as illustrated in Figure 1c.

### 2.2. Polyhedral Geometry Optimization

The neighboring polyhedra obtained from the Voronoi tessellation are in contact. Scaling down the aggregates will lead to parallel faces of neighboring aggregates, which differs from the real distribution of aggregates. If these polyhedra are used for finite element analysis, as the load increases, damage will occur simultaneously in the interfacial transition zones between all pairs of parallel faces, and the failure behavior of concrete cannot be captured accurately. Furthermore, Voronoi polyhedra often exhibit defects such as short edges and sharp angles, which may lead to convergence issues in nonlinear analysis and should be eliminated.

#### 2.2.1. Eliminating Short Edges

**Figure 2:**
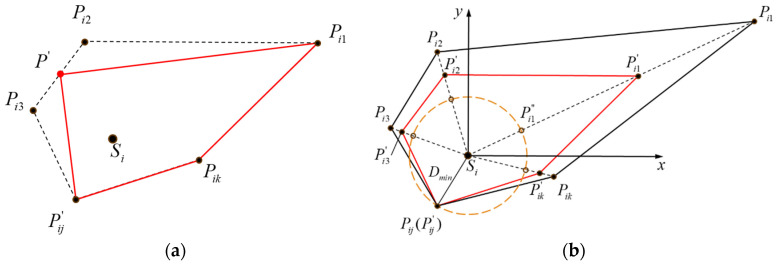
(**a**) Short-edge elimination and (**b**) non-uniform scaling approach.

To eliminate the short edges in Voronoi polyhedra, Xu et al. [27] conducted geometric optimization of “very small angles and edges” to obtain improved polyhedral shapes through a technique called “micro-cutting”. Naderi et al. [28] used the Loop subdivision surfaces technique to optimize the short edges and sharp angles in Voronoi polyhedra. Other researchers restricted the minimum distance between vertices [17,29] by using algorithms such as edge deletion to address geometric feature issues in polyhedra. Similarly, a new algorithm for optimizing short edges is proposed in this paper. Figure 2a shows a two-dimensional schematic of vertex merging for short edges. A user-defined minimum edge length is introduced. The ends of the edges with lengths smaller than the minimum edge length will be merged into a new vertex, and the number of edges will be decreased by one. The coordinates of the new vertex are calculated as the midpoint of the edge.

#### 2.2.2. Improving Sharp Angles

To improve the sharp angles in Voronoi polyhedra, a non-uniform scaling-down algorithm is conducted. Figure 2b shows a schematic illustration of the algorithm, taking a two-dimensional polygon as an example. The black polygon denotes the original, while the red one is the scaled-down form. Our strategy is to adapt different scaling factors based on the distance between vertices and seed points. For the vertices that are far from the seed point, the scaling factor of the distance needs to be relatively small, while for the vertices that are close to the seed point, the scaling factor can be a number close to one. The specific steps are as follows:

(1) Randomly select a polyhedron *V_i_*(*P_i_*, *S_i_*) and a vertex *P_i_*(*P_i_*_1_, *P_i_*_2_, …, *P_ik_*), where *k* represents the number of vertices.
(2) Calculate the distance *D_ij_* from the seed point to the vertices:
  (1) $$D_{ij}=d\left(P_{ij},\;S_{i}\right)\;\left(j=1,\;2,\;3,\;\ldots ,\;k\right)$$
(3) Find the minimum distance *D*_min_ = min(*d*(*P_ij_*, *S_i_*)).
(4) Calculate the distance *D_ij_′* from the seed point to the new vertices using the following expression:

(2) $$D_{ij}^{\prime }=D_{\min}+\Delta D_{ij}\times \beta$$

where Δ*D_ij_* is calculated by Δ*D_ij_* = *D_ij_* − *D*_min_, and *β* is the scaling factor between 0 and 1, which is set to 0.5 in Figure 2b.

(5) Perform non-uniform scaling down on the polyhedron, with the coordinates of the new vertices denoted as *P_i_′*(*P_i_*_1_*′*, *P_i_*_2_*′*, …, *P_ik_′*):
  (3) $$P_{ij}^{\prime }=S_{i}+q_{ij}\cdot \vec{\nu_{ij}}$$
  (4) $$q_{ij}=\frac{D_{ij}^{\prime }}{D_{ij}}$$
  (5) $$\vec{\nu_{ij}}=P_{ij}-S_{i}$$

**Figure 3:**
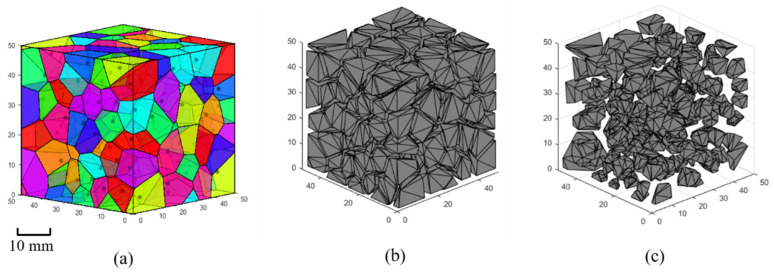
Improving parallel adjacent faces: (**a**) polyhedra generated by Voronoi tessellation, (**b**) polyhedra after eliminating short edges, and (**c**) polyhedra after improving sharp angles.

By applying the two operations described above to each polyhedron, a group of polyhedra with random shapes can be achieved, as shown in Figure 3, and the “convexity” of the polyhedra is preserved to the fullest extent.

### 2.3. Adjustment of Aggregate Grading

#### 2.3.1. Aggregate Grading

Concrete structure grading theory includes continuous grading and discontinuous grading. Based on continuous grading theory, the aggregate grading is calculated. The ideal grading curve proposed by Fuller and Thompson [30] is widely used and accepted.

The Fuller curve is defined as

(6) $$P\left(d\right)=100{\left(\frac{d}{d_{max}}\right)}^{n}$$

where *d* is the aperture of the sieve, *P*(*d*) represents the cumulative percentage passing the sieve, *d*_max_ represents the maximum size of the aggregate particles, and *n* is a constant parameter, which is typically set to 0.45–0.7. The formula for calculating the weight percentage of particles in the range [*d_i_*, *d_i_*_+1_] is [12]

(7) $$P\left(d_{i},\;d_{i+1}\right)=P\left(d_{i+1}\right)-P\left(d_{i}\right)$$

For aggregates distributed continuously in this particle size range, the volume calculation formula is [12]

(8) $$V_{\mathrm{agg}}\left(d_{i},\;d_{i+1}\right)=\int_{d_{i}}^{d_{i+1}}\frac{4}{3}\pi x^{3}dx=\frac{4}{3}\pi d_{i,\;i+1}^{3}\left(d_{i+1}-d_{i}\right)$$

where (*d_i_*_,*i*+1_) is the representative size of the grading range and can be calculated by

(9) $$d_{i,i+1}={\left(\frac{\left({d_{i}}^{2}+d_{i+1}^{2}\right)\left(d_{i}+d_{i+1}\right)}{4}\right)}^{\frac{1}{3}}$$

During aggregate simulation and placement, the grading of aggregates should be represented by the cumulative quantity percentage instead of the cumulative mass percentage. Assuming that the density of aggregates is constant, the cumulative quantity percentage can be calculated based on the cumulative mass percentage. The quantity percentage of the grading range [*d_i_*, *d_i_*_+1_] is

(10) $$Q\left(d_{i},\;d_{i+1}\right)=\frac{P\left(d_{i},\;d_{i+1}\right)/d_{i,\;i+1}^{3}}{\sum_{j=1}^{n}P\left(d_{j},\;d_{j+1}\right)/d_{j,\;j+1}^{3}}$$

The cumulative quantity percentage can be calculated by

(11) $$Q\left(d_{i+1}\right)=\sum_{j=1}^{i}Q\left(d_{j},\;d_{j+1}\right)$$

#### 2.3.2. Range Mapping Algorithm

The implementation of aggregate grading relies on the range mapping and uniform scaling of polyhedra. Range mapping refers to grouping the generated Voronoi polyhedra collection based on the particle size and determining appropriate scaling factors for each group based on their particle size. Uniform scaling of polyhedra means that, within a specific polyhedron, the distances between all vertices to the seed point are adjusted based on a uniform scaling factor.

The steps for range mapping are as follows:

(1) For a polyhedron *V_i_*(*P_i_*, *S_i_*) with *j* vertices, calculate the distance *d*(*P_im_*, *P_in_*) (*m* ≠ *n*; *m*, *n* = 1, 2, 3, …, *j*) between any two vertices *P_im_* and *P_in_*. The maximum distance is taken as the particle size of the polyhedron and denoted as *d*(*V_i_*) = max(*d*(*P_im_*, *P_in_*)).
(2) Sort the polyhedra by particle size from small to large and range the Voronoi polyhedra collection based on the number of polyhedra in each sub-range, which is calculated as follows:

(12) $$N\left(D_{i,\;1},\;D_{i,\;2}\right)=Q\left(d_{i},\;d_{i+1}\right)\times N_{poly}$$

where *N_poly_* is the number of Voronoi polyhedra, [*D_i_*_,1_, *D_i_*_,2_] is the particle size range, *D_i_*_,1_ is the minimum particle size of the *i*th range, and *D_i_*_,2_ is the maximum particle size of the range.

(3) Map [*D_i_*_,1_, *D_i_*_,2_] to [*d_i_*, *d_i_*_+1_]. *D_s_* represents the size of the polyhedron in the particle size range [*D_i_*_,1_, *D_i_*_,2_]. Any *D_s_* can be used to find the corresponding value *d_s_* in [*d_i_*, *d_i_*_+1_] according to
  (13) $$d_{s}=\frac{d_{i+1}-d_{i}}{D_{i,\;2}-D_{i,\;1}}\times (D_{s}-D_{i,\;1})+d_{i}$$
(4) Scale the polyhedral uniformly. The original vertex *P_i_* of a specific polyhedron moves toward the seed point *S_i_*, and the coordinates of the new vertex *P_i_′* are calculated according to a scaling factor *q_s_*, which is determined by
  (14) $$q_{s}=\frac{d_{i+1}-d_{i}}{D_{i,\;2}-D_{i,\;1}}\times \frac{D_{s}-D_{i,\;1}}{D_{s}}+\frac{d_{i}}{D_{s}}$$
  (15) $$\vec{\nu }=P_{i}-S_{i}$$
  (16) $${P_{i}}^{\prime }=S_{i}+q_{s}\cdot \vec{\nu }$$

### 2.4. Comparison with Fuller’s Curve

**Figure 4:**
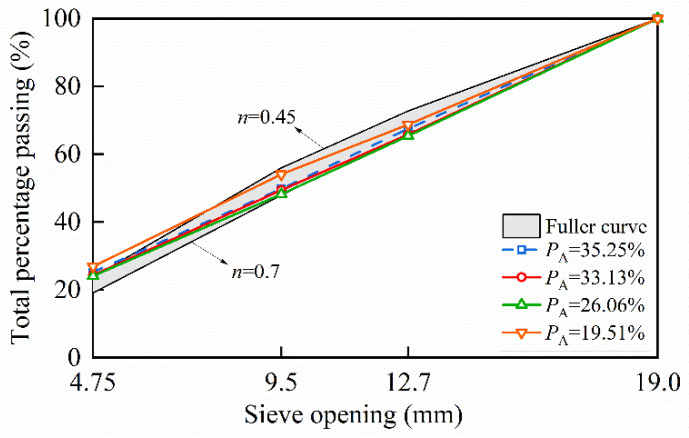
Fuller curves and grading curves of aggregate models with different volume fractions.

Using the grading type of aggregates from Table 1, the Fuller gradation curve was calculated based on Equations (6) and (7), and Fuller curves were plotted for *n* values ranging from 0.45 to 0.7, as shown in Figure 4. This range is indicated by the shaded blue area. Since the typical range for *n* is 0.45–0.7, it can be concluded that aggregate models with gradation curves within this range conform to Fuller’s gradation. Figure 4 displays the gradation curves of generated aggregate models with different volume fractions, where PA represents the aggregate volume fraction. It can be observed that the generated aggregate models generally align well with the Fuller curve. However, the cumulative passing percentage for smaller sieve sizes exceeds that of the Fuller curve due to the higher generation probability of smaller aggregates and the fact that the gradation adjustment algorithm starts with small aggregates. Additionally, larger aggregates are partially “cut” during polyhedral geometry optimization and gradation adjustment processes, which also contributes to the higher cumulative passing percentage for smaller aggregates.

### 2.5. Flowchart of the V-RW Algorithm

**Figure 5:**
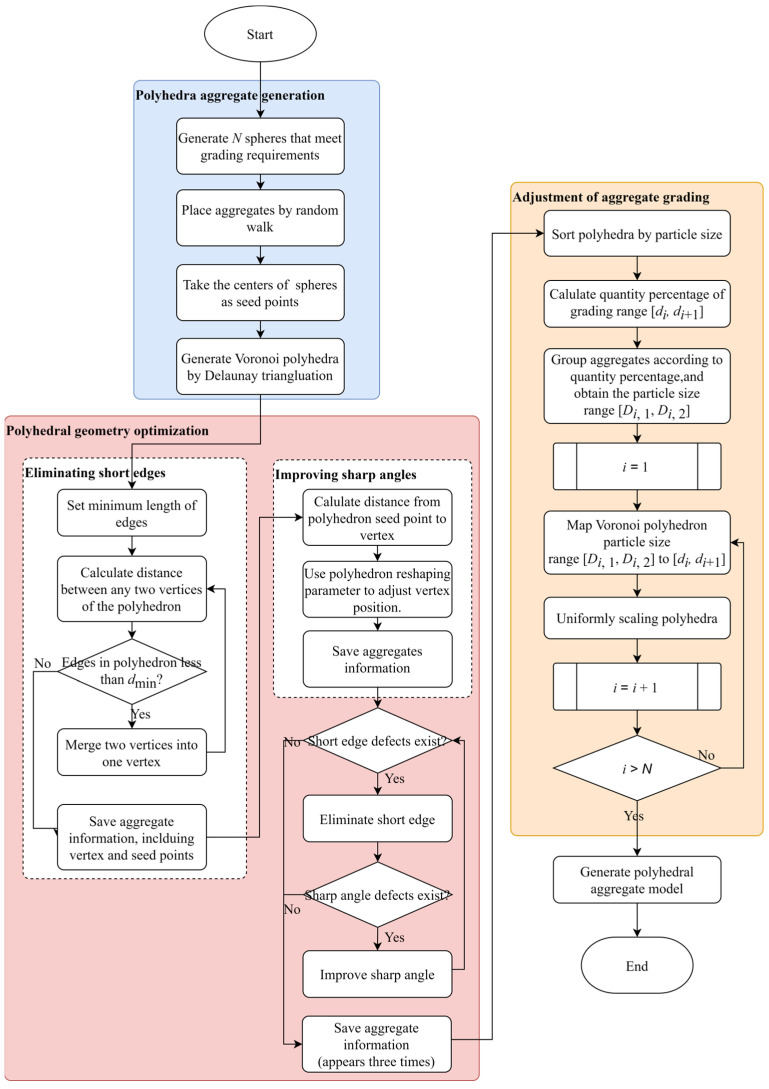
Flowchart of the Voronoi–random walk (V-RW) algorithm.

The V-RW algorithm consists of three steps, as shown in Figure 5.

Step 1: Voronoi polyhedra generation

The RWA is used to generate spheres that meet the grading requirements. The centers of these spheres are taken as seed points, and Delaunay triangulation is used to generate Voronoi polyhedra based on these seed points.

Step 2: Polyhedron geometry optimization, including short-edge elimination and sharp-angle improvement

In the short-edge elimination operation, a minimum length of the edges is preset. In the sharp-angle improvement operation, the distance from the polyhedron seed point to the vertex is obtained from Equation (1). Then, the scaling factor *β* is applied to adjust the positions of the vertices. The coordinates of the new vertices are obtained by Equations (2)–(5), thereby improving the sharp-angle defects. Sharp angles and short edges are iteratively checked to achieve polyhedron geometric optimization.

Step 3: Adjustment of aggregate grading

The particle size range [*d_i_*, *d_i_*_+1_] is used for standardized grading. The percentage of particles for each particle size range is determined based on Equations (10) and (12). The generated polyhedron aggregates are sorted by particle size and grouped according to the percentage determined above; the particle size range [*D_i_*_,1_, *D_i_*_,2_] is obtained. The scaling factor is calculated by Equation (14), the Voronoi polyhedron particle size range [*D_i_*_,1_, *D_i_*_,2_] is mapped to [*d_i_*, *d_i_*_+1_], and the coordinates of the new polyhedron vertices are obtained according to Equation (16).

## 3. Numerical Simulations

During the course of this study, a finite element (FE) model was designed using ABAQUS 2017, specifically tailored to the previously generated aggregate model, with the purpose of validating the damage scenarios within the mesostructure of concrete. The details of the FE modeling are discussed in the following sections.

### 3.1. Mesoscale Model of Aggregate

**Figure 6:**
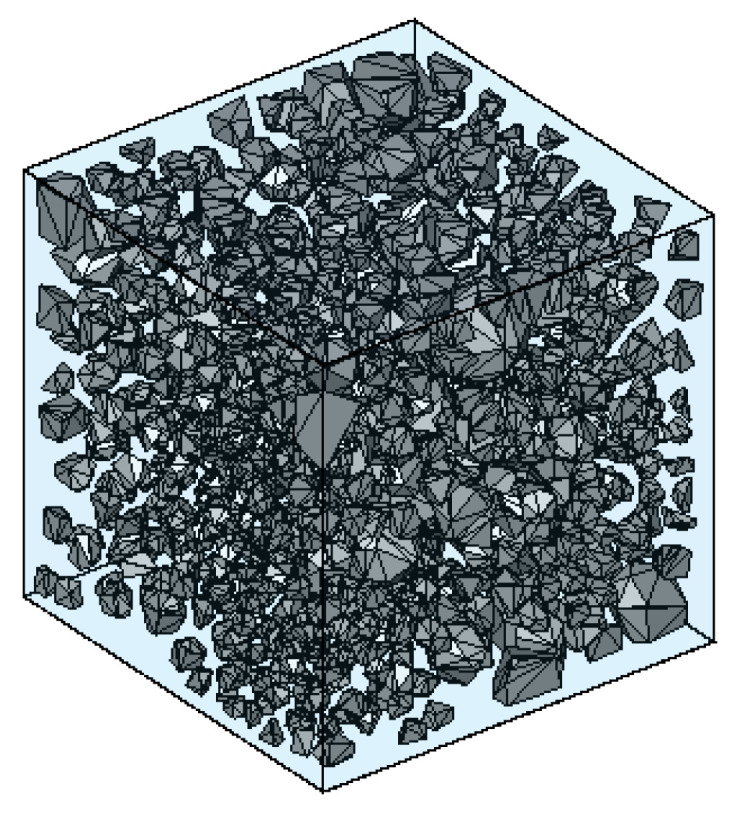
Polyhedral aggregate model.

In this paper, a target domain with the dimensions of 50 × 50 × 50 mm was used for aggregate generation. The size distribution is shown in Table 1. The parameter *n* for the Fuller curve was set as 0.6. For the geometric optimization of the polyhedra, the minimum length of the edge was set as 0.02 mm. To ensure the rationality of the aggregate model, it was necessary to generate as many seed points as possible in the target area. The model chosen for the simulation discussed in this section comprised 820 aggregate particles. Figure 6 shows the aggregate model with an aggregate volume fraction of 26.06%.

### 3.2. Finite Element Modeling

**Figure 7:**
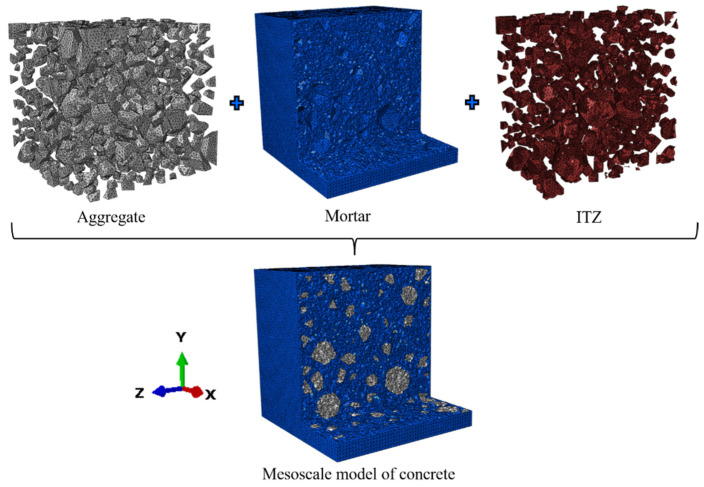
Finite element model of the mesoscale concrete.

The target domain of the mesostructure of concrete is a cube with a side length of 50 mm. The geometry and spatial distribution of the aggregate model were imported from MATLAB R2021b to ABAQUS 2017 for geometric modeling. In the example from Section 3.1, the aggregates were cut from a cube with a side length of 50 mm using Boolean operations, and the rest was defined as mortar. At the interfaces between the aggregates and mortar, cohesive elements with zero thickness were inserted [3,31]. Finally, the three components were assembled for the finite element modeling, as shown in Figure 7.

The element type of the aggregates and mortar was C3D4, and COH3D6 was used for the ITZ. It should be noted that the C3D4 element used in this study is a four-node tetrahedral element, which is known for its good performance in studying the internal components of concrete. The COH3D6 element, on the other hand, is a six-node wedge cohesive element that can represent the interface behavior between concrete and aggregates.

**Figure 8:**
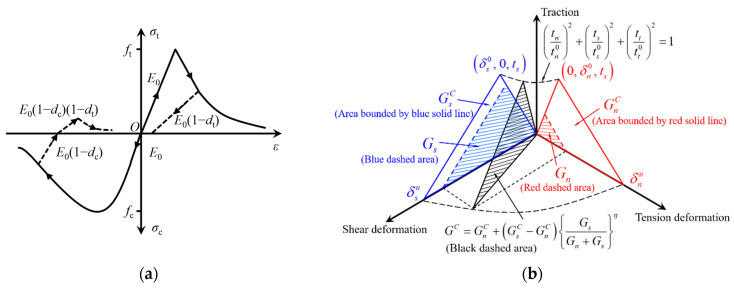
(**a**) Concrete damaged plasticity model and (**b**) quadratic nominal stress criterion and mixed-mode fracture criterion.

The concrete damaged plasticity model can accurately simulate the nonlinear behavior of mesoscale concrete [20,22,32]. The CDP model was applied to the mortar matrix, and the values of normal stress, tangential stress, and fracture energy in the damage evolution of the mortar matrix are all based on previous studies [32]. In this paper, the compressive strain hardening and softening curves follow GB50010 [33,34], and the constitutive relation is shown in Figure 8a. Additionally, the detailed CDP parameters are as follows: the dilation angle is 35°, plastic eccentricity is 0.1, the ratio of biaxial to uniaxial compressive strength is 1.16, the concrete yield shape parameter is 0.667, and the viscosity parameter is 0.0005 [20,32,35]. A bilinear constitutive law was used in cohesive elements due to its excellent computational efficiency and high accuracy [36]. The values of maximum nominal stress and fracture energy in damage evolution were taken from a previous study [31]. *G^C^* is mixed-mode fracture energy, *G*_n_ is strain energy in the normal direction, *G*_s_ is shear strain energies in two directions, and the mixed-mode fracture criterion is shown in Figure 8b. The main material properties of the mesoscale model are shown in Table 2.

During the simulation process, we conducted a mesh sensitivity validation on the mesoscale model presented in Figure 7, employing a global mesh with element sizes of 0.7 mm, 1 mm, 1.4 mm, and 2.1 mm, respectively. The analysis indicated that the 1 mm element size provided a good balance between computational efficiency and accuracy. Consequently, the global mesh size was set to 1 mm.

Finite element analysis was performed using the dynamic explicit method to simulate the uniaxial compression of the concrete cubic specimen. Loading was applied in the form of displacement, with two pads set in the Y direction of the specimen. The upper pad was subjected to a vertical displacement of 0.2 mm in the negative Y direction, while the lower pad was fixed. Guo et al. [37] believe that the friction coefficient can reflect the real experimental conditions within the range of 0.1 to 0.7. To simulate the failure mode of this concrete cubic specimen during compression, frictional constraints were set between the upper and lower pads and the contact surface with a friction coefficient of 0.6.

### 3.3. Simulation Results and Discussion

**Figure 9:**
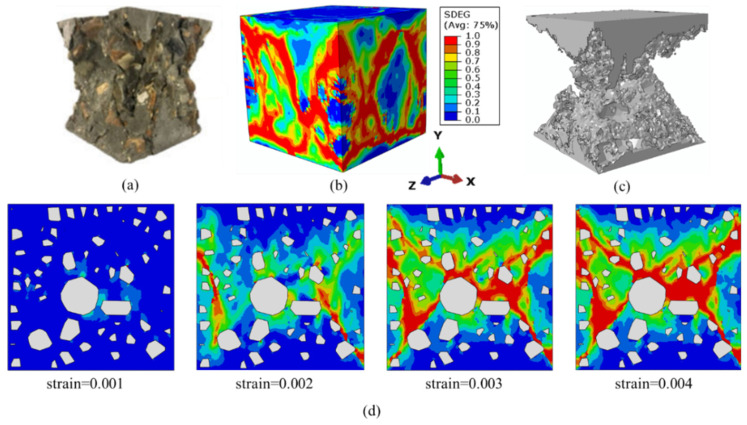
(**a**) Failure mode from the previous paper [20], (**b**) SDEG (scalar stiffness degradation variable) of the concrete model, (**c**) simulated failure mode, and (**d**) SDEG of the internal section.

The fracture process of the concrete cube is represented by the damage distribution of the mortar. “SDEG” represents the overall scalar stiffness degradation variable, with a range of 0 to 1, where 0 represents undamaged and 1 indicates completely damaged [4]. The value of SDEG increases with damage accumulation, and elements with an SDEG value greater than 0.9 are regarded as cracks [20,32]. Figure 9b shows the final damage distribution of a three-dimensional concrete cube. Figure 9a presents the failure mode observed in the experimental results from the previous paper [20]. During the failure process, the number of oblique cracks in the concrete cube increased, and triangular-shaped cracks appeared near the top and bottom regions, as shown in Figure 9b, where local triangular damage with wedge-shaped blocks is evident on the model surface. The results indicated that localized damage and detachment occurred, as shown in Figure 9c. To clearly display the final failure mode, the elements that had suffered damage and detachment were removed. Figure 9d shows the damage distribution at the internal section of the concrete cube, in which the white areas represent aggregates. It is observed that the high-stress regions were mostly concentrated around the aggregates, and cracks nucleated and propagated along narrow paths on the surfaces of the aggregates and within the mortar. This was because the ITZ was the weakest part of the concrete cube.

**Figure 10:**
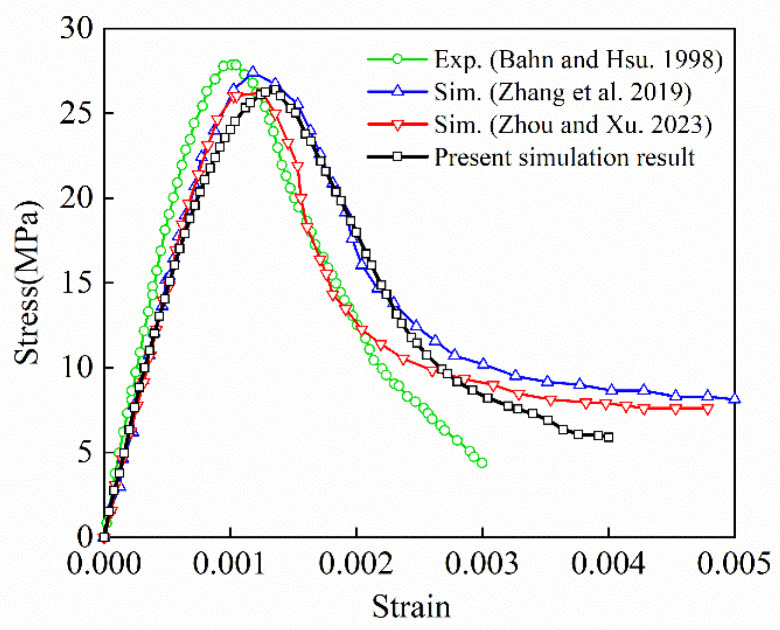
Stress–strain curves from experiments [38] and numerical simulations [29,32] in previous papers and the results of the present method.

The numerical results showed that damage was almost nonexistent at the top and bottom surfaces of the concrete cube but was observed in the middle of the model. The development of lateral tensile stress was limited by the tangential constraint, preventing cracks from propagating parallel to the loading direction. This is consistent with the results obtained under high-friction-coefficient conditions [29]. The overall failure mode of the concrete cube resembled an hourglass shape, similar to previous experimental results [20]. Figure 10 shows the stress–strain curves of the present method and from the literature [29,32,38]. From Figure 10, it can be observed that the curve simulated in this study exhibited a trend in the elastic stage, peak strength, and degradation stage that was consistent with the results in the literature overall. The differences in these curves may be attributed to factors such as the aggregate characteristics (e.g., shape, gradation, volume fraction, and random distribution), mesh properties (element type and size), and damage model employed.

## 4. Discussion

### 4.1. Efficiency of the V-RW Algorithm

All aggregate models were generated using MATLAB on an Intel Core i5@4.4 GHz desktop computer with 16 GB of RAM. Under these computational conditions, different methods were used to generate models with the same aggregate volume fraction in a cube with aggregate gradation *G*_I_, as shown in Table 3. Table 4 presents a comparison of the time consumption values for different methods of generating aggregates. When the aggregate volume fraction was 15%, the computational time of the V-RW algorithm was 55.6% lower than that of the random placement algorithm and 50.7% lower than that of the RWA. When the aggregate volume fraction was 20%, the results of the random placement algorithm were still valid, but the computational time was unacceptable. The V-RW could lower the computational time by 66.6% compared to that of the RWA. When the aggregate volume fraction was 25%, the V-RW could lower the computational time by 73.4% compared to that of the RWA.

Based on the above discussion, we can conclude that (1) the efficiency of the V-RW algorithm was much higher than those of the other two methods, and (2) it can be used for high aggregate volume fraction placement without a decrease in efficiency. This algorithm is more suitable for simulating concrete specimens with high aggregate volume fractions.

### 4.2. Polyhedral Reshaping Parameter

From Equation (5), it can be inferred that as *β* increases, the magnitude of the seed point movement decreases, and the scaled-down polyhedron shape is more similar to that of the original Voronoi polyhedron. This helps to increase the aggregate volume fraction. Table 5 shows the relationship between *β* and the aggregate volume fraction, where an increase in *β* led to a higher aggregate volume fraction.

In Section 2.2.2, we applied the same value of *β* for all aggregate particles, which partially addressed the issue of parallel adjacent faces. However, due to the random distribution of aggregates, there may still be small angles between adjacent faces. To fully resolve this issue, different values of *β* were applied for different aggregate particles, so that the scaling factor of the vertices toward seed points varied within the same aggregate. For this purpose, random numbers were generated within a certain range using a random number generator. Since we used uniformly distributed random numbers, *β* = 0.6 was replaced by *β* ∈ (0.2, 1). Table 6 shows the influence of random values on the aggregate volume fraction. It can be observed that randomly selecting *β* values could slightly increase the aggregate volume fraction and address the issue of parallel adjacent faces as well.

### 4.3. Aggregate Gradation

#### 4.3.1. Grading Type

**Figure 11:**
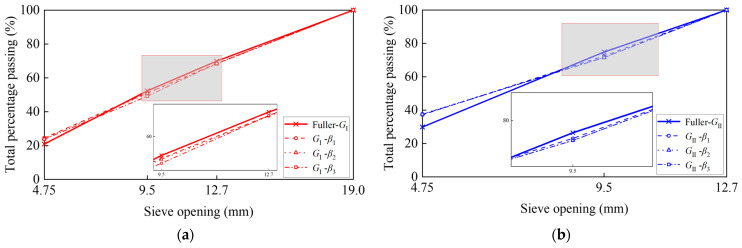
Grading comparison between simulated *G*_II_ curves with different scaling factors and the Fuller curve: (**a**) *G*_I_ and (**b**) *G*_II_.

To investigate the impact of the aggregate grading, we used the aggregate size distribution from the previous paper [39], as shown in Table 3, with one group using a four-level grading and the other using a three-level grading. The aggregate distribution was obtained based on the Fuller curve. Since the Fuller curve describes the ideal particle distribution, the simulated curve did not completely overlap with the Fuller curve but approximated it as closely as possible. Figure 11a,b show the comparison between the gradings of *G*_I_ and *G*_II_ aggregates and the Fuller curve. Overall, the grading curves of *G*_I_ and *G*_II_ were quite close to the Fuller curve. Specifically, the curve of *G*_I_ matched the Fuller curve well, while *G*_II_ exhibited a slightly larger deviation. However, both deviations were very small.

#### 4.3.2. Range Number in the Range Mapping Algorithm

**Figure 12:**
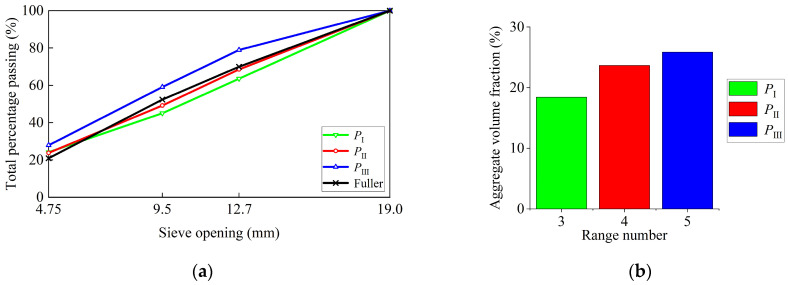
(**a**) Comparison between generated aggregate grading curves and the Fuller curve. (**b**) Aggregate volume fractions for different range numbers.

In the range mapping algorithm, the target particle size range (2.36, 19.0) was mapped and scaled into three, four, and five ranges. We analyzed the influences of the range numbers on the aggregate grading and volume fraction. Table 7 shows the sizes for each range. Figure 12a compares the grading curves of different range numbers with the Fuller curve. The grading curve of the five-range mapping deviated from the Fuller curve, with an excess of aggregates smaller than 12.7 mm and a shortage of aggregates larger than 12.7 mm. In contrast, the grading curve of the three-range mapping showed the opposite trend, with a shortage of aggregates smaller than 12.7 mm. The grading curve of the four-range mapping aligned well with the Fuller curve.

Figure 12b presents the aggregate volume fractions for different range numbers. As the number of ranges increased, the aggregate volume fraction also increased. Compared to *P*_I_, *P*_II_ showed a significant improvement in the aggregate volume fraction and better alignment with the Fuller curve. However, compared to *P*_II_, *P*_III_ showed a less significant improvement in the aggregate volume fraction and a larger deviation from the Fuller curve. Therefore, with a four-range mapping (*P*_II_), a well-aligned aggregate model with a higher volume fraction could be obtained.

## 5. Conclusions

This paper proposed a novel method for generating polyhedral aggregate models based on the random walk algorithm and Voronoi diagrams. The aggregate models were applied to finite element simulations of concrete cubes under uniaxial compression. The main conclusions were as follows:

(1) In the proposed method, the random walk algorithm was used to place seed points, which were then used to generate Voronoi polyhedra. The geometric shapes of these polyhedra were optimized through short-edge elimination and sharp-angle improvement. A range mapping algorithm is employed to obtain aggregate models that meet the grading requirements. In the simulations performed in this study, tetrahedral meshing was used to discretize aggregates and mortar, a cohesive element with zero thickness was used to simulate the ITZ, the concrete damaged plasticity model was used to simulate the damage in mortar, and a 3D mesostructure model of concrete was established.
(2) The grading of the aggregates affected the alignment with the Fuller curve. The generated four-level grading aggregate model aligned well with the Fuller curve. The polyhedral reshaping parameter *β* affected the aggregate volume fraction. Increasing *β* could improve the volume fraction. Changes in *β* had little effect on the aggregate grading curve. The range mapping algorithm could significantly improve the aggregate volume fraction as well, but too many or too few ranges could cause the grading curve to deviate from the Fuller curve. A four-range mapping could achieve a reasonable grading and a high aggregate volume fraction.
(3) The present algorithm has the following limitations: The generated aggregate particles all retain “convex” shapes, which do not align with the actual shapes of some aggregates with defects, particularly those with “flaky” and “needle-like” morphologies. During concrete damage, cracks tend to initiate and propagate from these defective aggregates due to stress concentration effects. Consequently, exploring the generation of aggregates with defective characteristics and conducting corresponding numerical simulations for validation have emerged as crucial directions for future research. On the other hand, in the current algorithm, the positions of all aggregate particles relative to their seed points remain fixed, which restricts the further increase in aggregate volume fraction to a certain extent. To address this issue, a potential research approach involves applying microrotations and translations to the aggregate particles, followed by the secondary generation of fine aggregate particles based on the adjusted coarse aggregates, aiming to generate aggregate models with higher volume fractions. Lastly, and most importantly, effectively extending the concrete mesoscale model to practical engineering applications poses a challenge. Integrating mesoscale models at the component level often leads to extremely large computational demands and high costs. Therefore, investigating the application of the proposed algorithm in numerical simulations of large-scale components has become a significant direction for future research.
(4) The V-RW algorithm showed a significant advantage of efficiently generating polyhedral aggregates with random shapes and specific grading models. Broadly, the V-RW method could also be used to simulate the mesostructures of other particle-filled composite materials, as well as the mesostructure of concrete composed of real aggregates. Examples of composite materials that consist of irregular polyhedral aggregates include crushed lightweight aggregate concrete, asphalt aggregate concrete, and coral lightweight aggregate concrete. Based on the established 3D mesostructure of concrete, finite element simulations can be used to study macroscopic mechanical properties and mesoscale mechanical behavior. With these advantages, the V-RW method provided an efficient and accurate tool for numerical simulations of concrete with real aggregates.

## Figures and Tables

**Table 1:** Particle size distribution of aggregates.

Sieve Size [mm]	Total Percentage Retained [%]	Total Percentage Passing [%]
19.0	0	100
12.7	24.37	75.63
9.5	49.43	50.57
4.75	65.79	34.21
2.36	100	0

**Table 2:** Material properties of the mesoscale model [31,32].

Material Properties	Aggregate	Mortar	ITZ
Density, *ρ* [kg/m^3^]	2800 ^	2000 ^	2000 *
Elasticity modulus, *E* [GPa]	70 ^	25 ^	-
Poisson ratio, *ν*	0.2 ^	0.2 ^	-
Compressive strength, *f*_c_ [MPa]	-	45 ^	-
Tensile strength, *f*_t_ [MPa]	-	4 ^	-
Fracture energy, *G*_f_ [N/mm]	-	0.06 ^	-
Cohesive stiffness, *E*/*E*_nn_, *G*_1_/*E*_ss_, and *G*_2_/*E*_tt_ [MPa/mm]	-	-	10^6^ *
Maximum nominal stress in normal direction, $t_{n}^{0}$ [MPa]	-	-	2.6 *
Maximum nominal stress in shear direction, $t_{s}^{0}$ and $t_{t}^{0}$ [MPa]	-	-	10 *
Normal mode fracture energy, $G_{n}^{C}$ [N/mm]	-	-	0.025 *
Shear mode fracture energy, $G_{s}^{C}$ [N/mm]	-	-	0.0625 *

Note: Data with “^” are quoted from Zhou [32]; data with “*” are quoted from Xiong [31].

**Table 3:** Grading types for analysis.

	Grading Type	
	*G*_I_	*G*_II_
Sieve size [mm]	19.0	-
	12.7	12.7
	9.5	9.5
	4.75	4.75
	2.36	2.36

**Table 4:** Comparison of time consumption values for different methods of generating aggregates.

Aggregate Volume Fraction	Time [s]		
	Random Aggregate by Grid Pre-Generation	Random Walk Algorithm	Voronoi–Random Walk Algorithm
15%	2073	1869	921
20%	78,992	3807	1273
25%	/	7701	2050
30%	/	/	2677
35%	/	/	3471
40%	/	/	4373

Note: “/” means that the time required by the aggregate model was not available. This may have been due to factors such as long computational times or algorithm performance limitations.

**Table 5:** Aggregate volume fractions for different polyhedral reshaping parameter values.

Polyhedral Reshaping Parameter *β*		*β*_1_	*β*_2_	*β*_3_
		0.6	0.5	0.4
Grading type	*G*_I_	26.06%	25.75%	23.65%
	*G*_II_	16.93%	16.22%	15.36%

**Table 6:** Comparison of aggregate volume fractions for different polyhedral reshaping parameters.

Polyhedral Reshaping Parameter *β*	0.6	(0.2, 1)	0.8	(0.6, 1)
Aggregate volume fraction	26.06%	26.64%	27.20%	29.29%

**Table 7:** Range types for analysis.

	Range Type		
	*P*_I_	*P*_II_	*P*_III_
Range size [mm]	19.0	19.0	19.0
	9.5	12.7	12.7
	4.75	9.5	9.5
	2.36	4.75	8
	-	2.36	4.75
	-	-	2.36

## Data Availability

The original contributions presented in the study are included in the article; further inquiries can be directed to the corresponding author.

## Nomenclature

*N*	Number of Voronoi seeds
*S_i_*	Coordinates of seed point
*P_i_*	Set of vertex coordinates of the *i*th Voronoi polyhedron
*V_i_*	Points set of the *i*th Voronoi polyhedron
*D_ij_*	Distance between *S_i_* and *P_ij_*
*D*_min_	Minimum distance between *S_i_* and *P_ij_*
*β*	Polyhedral reshaping parameter
*P_i_′*	Coordinates of the new vertices
*q*	Scaling factor
*d*	Aperture of the sieve
*P*(*d*)	Cumulative percentage passing the sieve
*d*_max_	Maximum size of the aggregate particles
*n*	A constant parameter
*V_agg_*	Volume of fraction of aggregates
*d*_*i*,*i*+1_	Representative size of the grading range
*Q*	Quantity percentage of the grading range
*N_poly_*	Number of Voronoi polyhedra
*D*_*i*,1_	Minimum particle size of the ith range
*D*_*i*,2_	Maximum particle size of the ith range
*D_s_*	Size of the polyhedron aggregate
*G*	Grading type
*P*	Range types
